# Diagnostic Utility of Relative Apical Sparing Index in Cardiac Amyloidosis Subtypes: A Comparative Study of Immunoglobulin Light Chain and Transthyretin Amyloid Cardiomyopathy

**DOI:** 10.1111/echo.70087

**Published:** 2025-01-28

**Authors:** Yoshihito Saijo, Hirotsugu Yamada, Natsumi Yamaguchi, Susumu Nishio, Robert Zheng, Tomonori Takahashi, Tomoya Hara, Muneyuki Kadota, Yutaka Kawabata, Rie Ueno, Tomomi Matsuura, Takayuki Ise, Koji Yamaguchi, Shusuke Yagi, Takeshi Soeki, Tetsuzo Wakatsuki, Masataka Sata

**Affiliations:** ^1^ Cardiovascular Department Tokushima University Hospital Tokushima Japan; ^2^ Department of Community Medicine for Cardiology Tokushima University Graduate School of Biomedical Sciences Takamatsu Japan; ^3^ Ultrasound Examination Center Tokushima University Hospital Tokushima Japan

**Keywords:** amyloidosis, apical sparing, cardiac function, speckle tracking echocardiography, strain

## Abstract

**Background:**

Speckles tracking echocardiography imaging enables clinicians to detect subtle systolic dysfunction. The aim of the present study was to elucidate the differences in speckle tracking echocardiographic findings between immunoglobulin light chain amyloid cardiomyopathy (AL‐CM) and transthyretin amyloid cardiomyopathy (TTR‐CM).

**Methods:**

The patients with a confirmed diagnosis of cardiac amyloidosis through cardiac biopsy from March 2013 to October 2022 were included. The relative apical sparing index (RASI) was calculated using speckle tracking echocardiography by the following equation; average apical strain/(average basal strain + mid strain).

**Results:**

The final study population consisted of 35 patients with cardiac amyloidosis (AL‐CM: 10 patients, TTR‐CM: 25 patients). The mean age was 74 ± 12 years. Although both subgroups had a gradual change of strain values from basal to apical segments, RASI was significantly lower in AL‐CM compared to TTR‐CM (0.92 ± 0.29 vs. 1.46 ± 0.53, *p* = 0.001). A RASI cutoff value of <1.0 proved useful in differentiating the diagnosis of AL‐CM from TTR‐CM (sensitivity: 81%, specificity: 70%, AUC: 0.82). A significant positive correlation with left ventricular mass index and RASI was found in AL‐CM, but not in TTR‐CM.

**Conclusion:**

The apical sparing phenomenon was more remarkable in TTR‐CM compared with AL‐CM. RASI might be useful for the discrimination of cardiac amyloidosis subtypes. There was a difference in the relationship of RASI with left ventricular wall thickness between the cardiac amyloidosis subtypes.

## Graphical abstract

Apical sparing phenomenon was more remarkable in transthyretin amyloid cardiomyopathy (TTR‐CM) than amyloid cardiomyopathy (AL‐CM). There was a significant difference in the relationship of apical sparing phenomenon with hypertrophy between TTR‐CM and AL‐CM.

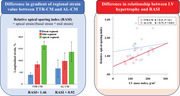

**Abbreviations**
AL‐CM: immunoglobulin light chains amyloid‐cardiomyopathy
ANCOVA: analysis of covariance
LS: longitudinal strain
LV: left ventricular
LVMI: left ventricular mass index
RASI: relative apical sparing index
TTR‐CM: transthyretin amyloid‐cardiomyopathy

## 1 Introduction

Amyloidosis increasingly recognized group of disorders characterized by the extracellular aggregates of insoluble beta‐fibrillar protein deposits within various organs [1]. Amyloid cardiomyopathy is mainly caused by two main subtypes of amyloidosis; immunoglobulin light chains amyloid‐cardiomyopathy (AL‐CM) and transthyretin amyloid‐cardiomyopathy (TTR‐CM) [2]. It is crucial to differentiate between AL‐CM and TTR‐CM from the perspective of treatment and prognosis [2, 3]. The differences of the conventional echocardiographic parameters between AL‐CM and TTR‐CM have been reported, patients with TTR‐CM having thicker left ventricular (LV) wall and lower LV ejection fraction compared with AL‐CM [4]. Recently speckles tracking echocardiography imaging enables clinicians to detect subtle systolic dysfunction in a variety of cardiac myopathy [5]. Given the difference in prognosis and geometry between AL‐CM and TTR‐CM, there may be markedly differences in speckle‐tracking echocardiographic findings between the two cardiac amyloidosis subtypes. We hypothesized that detecting subtle cardiac dysfunction using speckle tracking echocardiography may be more useful in distinguishing the two groups than conventional echocardiographic findings [6]. Hence, the aim of the present study was to elucidate the differences in speckle tracking echocardiographic findings between AL‐CM and TTR‐CM, and to assess whether it was useful for discrimination between the two cardiac amyloidosis subtypes.

## 2 Methods

### 2.1 Study Design and Population

The consecutive patients diagnosed as AL‐CM and TTR‐CM amyloidosis (wild type) through endomyocardial biopsy and pathological assessment from March 2013 to October 2022 were included in the present study, from patients with suspected cardiac amyloidosis with increased LV hypertrophy or amyloidosis deposits in other organs. We excluded patients with no echocardiographic assessment within 1‐month from myocardial biopsy. Additionally, patients with no‐pathophysiological assessment to prove amyloid deposit on cardiac muscle were excluded. Patients with hereditary TTR amyloidosis and amyloid‐A amyloidosis were excluded due to the small number. We obtained approval from the Institutional Ethics Committee of the Tokushima University.

### 2.2 Diagnosis of Amyloid Subtype

Amyloid typing was obtained by immune electron microscopy on formalin‐fixed paraffin‐embedded blocks after dewaxing and resin‐embedding. Selected sections were then processed for post‐embedding immunogold. TTR‐CM wild type was diagnosed based on the absence of a TTR gene mutation.

### 2.3 Echocardiographic Assessment

All patients underwent comprehensive echocardiograms using commercial instruments (GE Healthcare, Chicago, IL, USA, E9 and E95) by experienced investigators. The extracted conventional echocardiographic parameters included LV volumes and LV ejection fraction by Simpson's method, left ventricular mass index (LVMI), relative wall thickness, left atrial volume index, right ventricular systolic pressure [7], the ratio between early mitral flow velocity, and the average of the lateral and mitral annulus velocities (E/e’) referring to American society of echocardiography guidelines [8].

### 2.4 Speckle Tracking Echocardiography

2D‐speckle tracking echocardiography was utilized to characterize longitudinal systolic strain. Images were acquired at ≥40 frames/s at end‐expiration in apical long, two‐ and four‐chamber views (images with <40 frames/s were excluded as inadequate images) and analyzed using EchoPAC version 204 (GE Healthcare, Chicago, IL, USA). For each of the individual apical views, tracking was visually inspected throughout the systole to ensure adequate border tracking, and the endocardial contours were adjusted manually when necessary. The LV wall was divided into 17 segments (six segments in the basal segment, six segments in midsegments, and five segments in the apical segments), and the mean of each peak negative strain value of all segments was considered global longitudinal strain (LS). The LS values were reported as absolute values. The relative apical sparing index (RASI) was calculated by the following equation; average apical LS/(average basal LS + mid LS) [9].

### 2.5 Statistical Analysis

Continuous variables were expressed as mean ± standard deviation for normal distribution, or median and interquartile ranges for skewed distribution. Comparisons of variables between AL‐CM and TTR‐CM were performed with paired *t*‐test. Categorical data were expressed as number and percentage, and compared using the Chi‐square test or Fisher's exact test, as appropriate. Spearman's correlation coefficient was used to test associations between continuous variables. To assess whether these findings reflect the impact of amyloidosis subtypes, or are reflective of underlying LV geometry (LVMI), we performed an analysis of covariance (ANCOVA). The sensitivity and specificity of RASI for the discrimination of amyloidosis types were assessed using receiver operating characteristics curves. Youden's test was done to determine the accuracy of RASI in distinguishing AL‐CM from TTR‐CM and establish a cutoff value. To assess the relationship of variables with AL‐CM, we performed univariate and multivariate logistic regression analyses. Multivariable logistic regression analyses were performed using variables with a significant relationship in the univariable analyses, in addition to RASI. Some models were made due to a small number of the patients in the present study. Odds ratios with 95% confidence intervals were reported. Statistical analysis was performed using SPSS version 25 (SPSS Inc., Chicago, IL, USA). A *p* value of <0.05 was considered significant.

## 3 Results

### 3.1 Study Population

From 239 patients with suspected cardiac amyloidosis who underwent screening assessment for cardiac amyloidosis, 184 patients were diagnosed with other cardiomyopathies, and three patients with no pathophysiological assessment due to patient preferences. The remaining 52 patients, one patient with inherently TTR‐amyloidosis, and the other patient with amyloid‐A amyloidosis were excluded from the analysis. The final study population consisted of 50 patients with a confirmed diagnosis of cardiac amyloidosis, 21 patients having AL‐CM, and 29 patients with TTR‐CM. All patients underwent pathophysiological assessment and genetic tests to diagnosis amyloidosis types. From 50 patients, 15 patients were excluded due to suboptimal images, and finally speckle‐tracking strain evaluation was available in 35 patients (*n* = 10 in AL‐CM, *n* = 25 in TTR‐CM).

### 3.2 Clinical Characteristics

The baseline characteristics of the study groups are described in Table 1. The mean age was 72 ± 11 years, 35 patients (70%) were male. Patients with AL‐CM were younger than TTR‐CM patients (66 ± 10 vs. 78 ± 7 years, *p* < 0.001). In 50 patients as shown in Table S1, lower blood pressure, serum hemoglobin, serum total protein, and serum albumin were found in AL‐CM, when compared to TTR‐CM (respectively, *p *< 0.05).

**TABLE 1:** Clinical characteristics.

	TTR‐CM (*n* = 25)	AL‐CM (*n* = 10)	*P*
Age (years)	78 ± 8 (range 63–90)	65 ± 11 (range 48–80)	<0.001
Male (%)	18 (72)	5 (50)	0.23
Body mass index (kg/m^2^)	1.60 ± 0.19	1.60 ± 0.18	0.46
Heart rate (bpm)	71 ± 14	73 ± 12	0.36
Systolic blood pressure (mm Hg)	124 ± 23	102 ± 18	0.006
Diastolic blood pressure (mm Hg)(	71 ± 15	60 ± 13	0.022
Hypertension (%)	6 (24)	1 (10)	0.64
Diabetes mellitus (%)	2 (8)	2 (20)	0.56
Coronary artery disease (%)	2 (8)	0 (0)	1.0
White blood cell (×10^3^/mL)	6.4 ± 1.8	6.0 ± 1.2	0.25
Serum hemoglobin (g/dL)	14.1 ± 1.8	13.0 ± 1.6	0.049
Plate (×10^5^/mL)	224 ± 68	222 ± 58	0.46
Total serum protein (g/dL)	7.2 ± 0.5	6.0 ± 0.8	<0.001
Serum albumin (g/dL)	4.0 ± 0.4	3.6 ± 0.4	0.004
Serum uremia acid (mg/dL)	6.1 ± 1.6	6.9 ± 1.7	0.23
eGFR (mL/min/1.73m^2^)	53 ± 19	56 ± 19	0.37
Creatinine kinase (IU/L)	163 ± 92	111 ± 57	0.051
BNP (pg/mL)	265 (187–386)	1009 (247–1809)	0.045

*Note*: Normally distributed data are presented as mean ± SD, whereas non‐normally distributed data are presented a median (25th–75th percentiles).

Abbreviations: AL‐CM, immunoglobulin light‐chain amyloid cardiomyopathy; ATTR‐CM, transthyretin amyloid cardiomyopathy; BNP, B‐type natriuretic peptide; eGFR, estimated glomerular filtration rate.

### 3.3 Conventional Echocardiography Characteristics

All patients underwent a conventional echocardiography study. Detailed 2D‐echocardiography data are shown in Tables 2 and S2. In 50 patients, AL‐CM had smaller LV end‐diastolic volume (78 ± 24 vs. 93 ± 31 mL, *p *= 0.041), LV end‐systolic volume (31 ± 13 vs. 43 ± 21 mL, *p* = 0.014) and higher LV ejection fraction (60 ± 8 vs. 54 ± 10%, *p *= 0.014), compared to TTR‐CM. E/A and E/e’ in AL‐CM were likely to be higher than those in TTR‐CM, but not significant. There was a significant difference in LVMI, AL‐CM having a lower LVMI compared with TTR‐CM (125 ± 49 vs. 152 ± 39 g/m^2^, *p* = 0.015).

**TABLE 2:** Echocardiographic characteristics.

	TTR‐CM (*n* = 25)	AL‐CM (*n* = 10)	*p*
IVS wall thickness (mm)	13.8 ± 2.3	12.6 ± 2.5	0.099
Posterior wall thickness (mm)	13.0 ± 2.4	12.3 ± 2.9	0.23
LV mass index (g/m^2^)	155 ± 41	125 ± 53	0.13
Relative wall thickness	0.58 ± 0.13	0.61 ± 0.15	0.32
Max inferior vena cave (mm)	13 ± 5	14 ± 5	0.20
LV end‐diastolic volume (mL)	94 ± 33	83 ± 31	0.19
LV end‐systolic volume (mL)	45 ± 22	35 ± 17	0.12
LV ejection fraction (%)	53 ± 10	59 ± 8	0.071
LA volume index (mL/m^2^)	45 ± 14	46 ± 13	0.46
TR‐PG (mm Hg)	28 ± 8	31 ± 8	0.17
E/A ratio	1.80 ± 1.35	2.41 ± 1.14	0.12
E/e’ ratio	18.0 ± 8.5	22.9 ± 9.2	0.078

*Note*: Normally distributed data are presented as mean ± SD.

Abbreviations: AL‐CM, immunoglobulin light‐chain amyloid cardiomyopathy; ATTR‐CM, transthyretin amyloid cardiomyopathy; IVS, interventricular septum; LA, left atrial; LV, left ventricular; TR‐PG, tricuspid regurgitation‐pressure gradient.

### 3.4 Speckle Tracking Echocardiographic Characteristics

**FIGURE 1:**
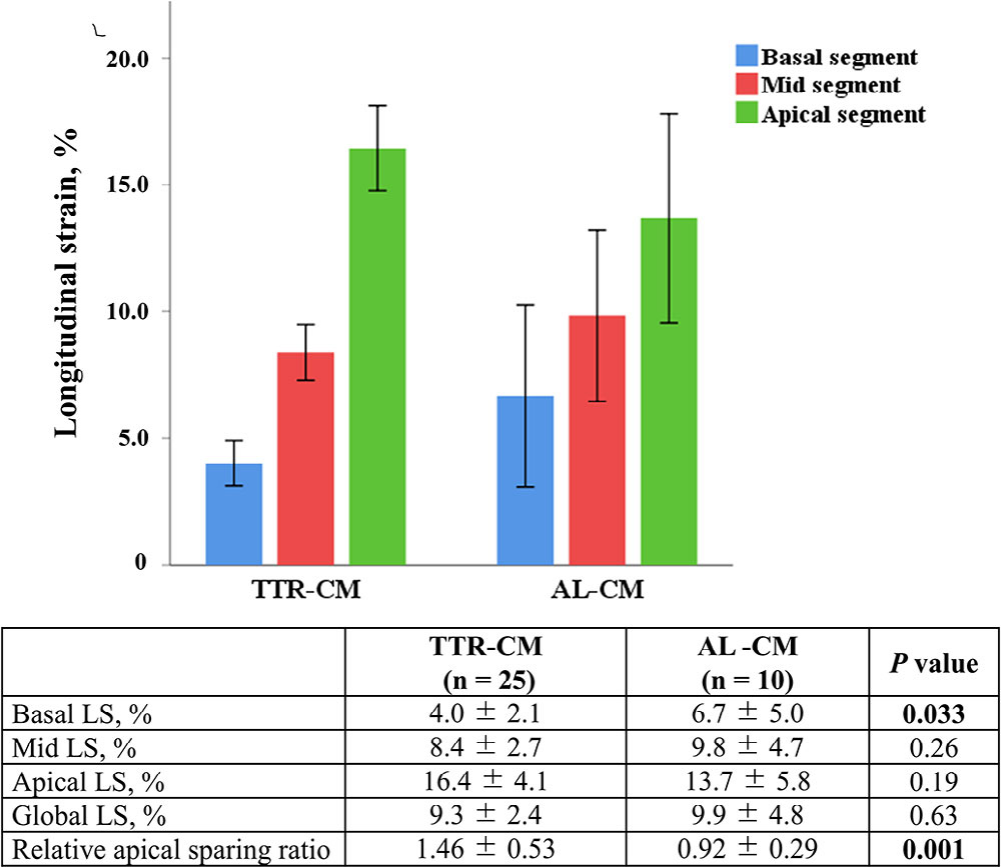
Variations of regional LV deformation. Longitudinal strain values in each three LV segments in AL‐CM (A) and TTR‐CM (B). AL‐CM, immunoglobulin light chain cardiomyopathy; LV, left ventricular; TTR‐CM, transthyretin amyloid cardiomyopathy.

**FIGURE 2:**
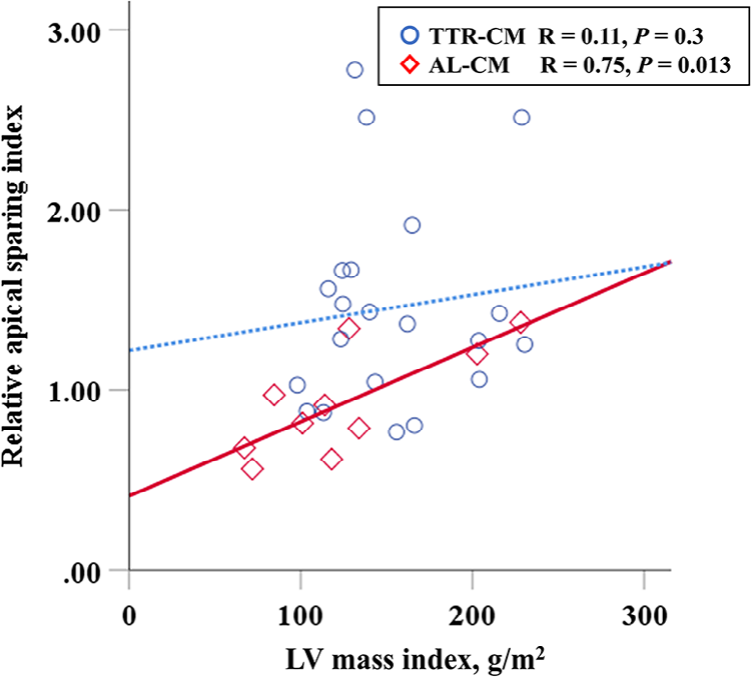
Correlation between relative apical sparing index and LV mass index in AL‐CM and TTR‐CM. AL‐CM, immunoglobulin light chain cardiomyopathy; LV, left ventricular; TTR‐CM, transthyretin amyloid cardiomyopathy.

AL‐CM and TTR‐CM revealed a similar impaired global LS (−9.9 ± 4.8 vs. −9.3 ± 2.4%, *p* = 0.63). As shown in Figure 1, although regional myocardial deformation analysis shows apical sparing phenomenon in both the amyloidosis subtypes, TTR‐CM had significantly a lower basal LS and higher RASI, when compared with AL‐CM (0.92 ± 0.29 vs. 1.46 ± 0.53, *p* = 0.001). There was a significant positive correlation of RASI with LVMI in AL‐CM, but not in TTR‐CM (Figure 2). After adjusting for LVMI in ANCOVA model, the difference in RASI between the two subtypes remained statistically significant *(p* = 0.02).

### 3.5 Diagnostic Value of RASI for Discrimination of Amyloidosis Subtypes

**FIGURE 3:**
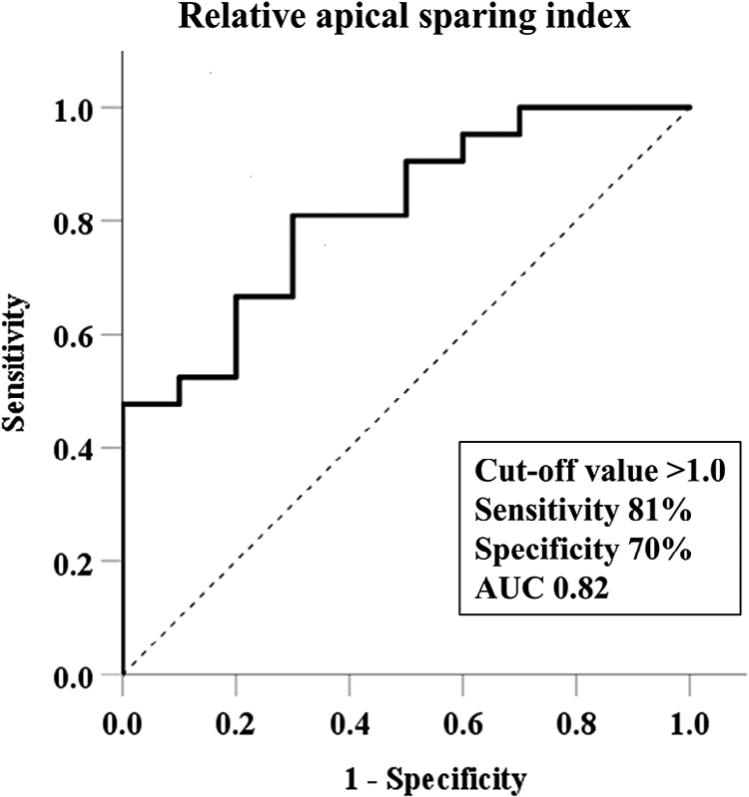
Receiver operating characteristics curve of relative apical sparing index for the detection of AL‐CM from TTR‐CM. AL‐CM, immunoglobulin light chain cardiomyopathy; TTR‐CM, transthyretin amyloid cardiomyopathy.

Receiver operating characteristics curve analysis showed an optimal cutoff value of 1.0 in RASI for the discrimination of AL‐CM from TTR‐CM (sensitivity 81%, specificity 70%, AUC 0.82, Figure 3). To assess the relationship of echocardiographic variables with AL‐CM, we performed univariate logistic regression analyses. As shown in Table 3, univariate logistic analysis shows that younger age (odds ratio: 0.83, 95% CIs: 0.74–0.92, *p* = 0.001), lower LVMI (odds ratio: 0.98, 95% CIs: 0.97–0.99, *p* = 0.039), smaller LV end‐systolic volume (odds ratio: 0.95, 95% CIs: 0.91–0.99, *p* = 0.039), higher LV ejection fraction (odds ratio: 1.09, 95% CIs: 1.00–1.18, *p* = 0.04) and RASI < 1.0 (odds ratio: 6.0, 95% CIs: 1.20–30.0, *p* = 0.029) were significantly associated with AL‐CM. Multivariate analyses reveal that RASI <1.0 remained a significant predictor for AL‐CM, even after adjusting conventional echocardiographic variables except age.

**TABLE 3:** Univariate and multivariate logistic regression analyses for diagnosis of AL‐CM.

	Univariate		Multivariate					
			Model 1		Model 2		Model 3	
Variables	Odds ratio (95% CIs)	*P*	Odds ratio (95% CIs)	*P*	Odds ratio (95% CIs)	*P*	Odds ratio (95% CIs)	*p*
Age	0.83 (0.74–0.92)	0.001	0.86 (0.76–0.98)	0.021				
Male	1.93 (0.57–6.58)	0.29						
LV mass index	0.98 (0.97–0.99)	0.039						
LV end‐diastolic volume	0.98 (0.96–1.00)	0.09						
LV end‐systolic volume	0.95 (0.91–0.99)	0.039			0.98 (0.94–1.03)	0.30		
LV ejection fraction	1.09 (1.00–1.18)	0.040					1.07 (0.96–1.19)	0.23
BNP	1.00 (1.00–1.002)	0.074						
RASI < 1.0	6.0 (1.20–30.0)	0.029	5.28 (0.76–36.8)	0.093	5.78 (1.12–29.9)	0.036	6.27 (1.18–33.4)	0.01

Abbreviations: AL‐CM, immunoglobulin light‐chain amyloid cardiomyopathy; BNP, B‐type natriuretic peptide; LV, left ventricular; RASI, relative apical sparing index.

## 4 Discussion

The present study using speckle tracking analysis adds new information on the differences in cardiac functional characteristics between AL‐CM and TTR‐CM; (1) the apical sparing phenomenon was more remarkable in TTR‐CM, when compared to AL‐CM; (2) there was a difference in the relationship between RASI and LVMI, where RASI was getting higher as myocardial hypertrophy worsened in AL‐CM while no association was found in TTR‐CM; and (3) RASI proved effective for discriminating between AL‐CM and TTR‐CM, with a cutoff value of 1.0 for the RASI.

Cardiac involvement in systematic amyloidosis is a determinant for prognosis due to several adverse cardiac events [10]. Previous studies demonstrate a remarkable difference in prognosis between AL‐CM and TTR‐CM, especially untreated AL amyloidosis associated with a median survival of approximately 6 months in patients with heart failure [11, 12]. Additionally, while chemotherapy treatment for the underlying disease is available in patients with AL‐CM, a recent large multicenter controlled trial has revealed that tafamidis improves prognosis and quality of life in patients with TTR amyloidosis by delaying disease progression [13, 14]. Therefore, discrimination in cardiac amyloidosis subtypes is crucial for selecting appropriate treatment and follow‐up care [15]. However, a correct diagnosis of amyloidosis subtypes often takes over 6 months because of the complexity of the diagnostic processes, including blood tests, multimodality imaging, biopsies, and genetic tests [16].

### 4.1 Role of Conventional Echocardiography for Cardiac Amyloidosis

Two‐dimensional (2D) echocardiography is a cornerstone for the identification of patients with suspected cardiac amyloidosis [2]. It plays a pivotal role in the diagnostic pathway of cardiac amyloidosis due to non‐invasive and popular imaging examinations. Some previous papers have reported the differences in the conventional echocardiographic findings between AL‐CM and TTR‐CM. TTR‐CM have higher LVMI and worse LV ejection fraction [11, 17]. These findings were in line with the present study. However, a more sensitive indicator with a high index of suspicion is needed to achieve prompt and accurate diagnosis of cardiac amyloidosis subtypes [18].

### 4.2 Difference of Longitudinal Strain Between AL‐CM and TTR‐CM

The present study demonstrated that both AL‐CM and TTR‐CM exhibit a base‐to‐apex gradient of LS. This phenomenon, first described by Phelan et al., is linked to thicker LV wall in basal to mid‐ventricular segments compared to the apex in AL amyloidosis patients [9]. In a study with 26 patients with AL‐CM and 17 patients with wild type TTR‐CM, reported by Ternacle et al., the differences in cardiac function including LS among cardiac amyloidosis subtypes are observed [19]. This study is congruent with the present study in showing that global LS value was similarly impaired in AL‐CM and wild‐type‐TTR‐CM, and base‐to‐apex gradient is found in both AL‐CM and TTR‐CM. Additionally, the unique point of the present study is that a significant difference in RASI between TTR‐CM and AL‐CM was observed. A few previous papers show that there is no difference in RASI between AL‐CM and TTR‐CM [9, 20]. This discrepancy might be attributed to differences in study populations. When compared with the previous paper reported by Phelan et al. (mean LV wall thickness; 16.9 ± 2.8 mm, and LVMI; 149 ± 41 g/m^2^), the subjects in the present study had thinner LV wall thickness of 13.1 ± 2.2 mm and lower LVMI of 141 ± 44 g/m^2^). Due to the positive correlation of RASI with LV wall thickness in AL‐CM, the significant difference in RASI between AL‐CM and TTR‐CM might be generated at early stage of amyloidosis.

The underlying mechanism of the differential relationship between LVMI and RASI remains unclear, but may involve differences in amyloid accumulation patterns and disease stages. Gaspari et al. report that the relationship of myocardial LS with pathophysiological assessment of amyloid burden quantification from autopsy [20]. Although the correlation between LS in each segment and segmental amyloid deposit is found, the difference in amyloid burden between amyloidosis subtypes has not been assessed. To clarify these clinical questions, a further study with pathological assessment is needed.

### 4.3 Utility of RASI for Discrimination of Cardiac Amyloidosis

RASI has been reported as a useful indicator for discriminating hypertrophic cardiac diseases [9]. A cutoff value of 1.0 for RASI in the present study aligns with values reported for discrimination of cardiac amyloidosis from other cardiac diseases with wall thickness [9]. Of note, some AL‐CM patients with thinner LV walls exhibited RASI below 1.0 in the present study. In other word, AL amyloidosis without LV hypertrophy at the early stage may not be ruled out even in the absence of a typical apical sparing phenomenon.

### 4.4 Clinical Implication

Echocardiographic evaluation using RASI may contribute to expanding the accuracy of cardiac amyloidosis subtype identification by increasing pre‐test probability. Although the definitive diagnosis of amyloidosis subtype requires a pathophysiological assessment, timely therapeutic interventions enabled by improved diagnostic accuracy could enhance patient's outcomes. Further multicenter research with larger cohorts is warranted to confirm the utility of these findings from the present study in cardiac amyloidosis diagnosis and management. The present study including the early stage of AL amyloidosis is more applicable to clinical practice because early diagnosis and therapeutic intervention can improve cardiac function and prognosis in AL amyloidosis [21].

Variability in the diagnostic accuracy of apical sparing phenomenon for distinguishing cardiac amyloidosis from other cardiac myopathies with hypertrophy, as reported recently, may be attributed to the relationship between base‐to‐apex gradient of LS value and LV wall thickness in AL‐CM [22].

### 4.5 Limitations

This study has several limitations. It was a single‐center study with a relatively small sample size for each amyloidosis subtype. The present study excluded the patients with other causes of cardiac amyloidosis due to small number. All patients had not underwent cardiac magnetic resonance imaging. We were unable to perform sufficient multivariate logistic regression analysis due to a small number of cohorts. Therefore, it is possible that the predictive ability of RASI has been underestimated. Stain value is a sensitive marker of myocardium systolic function, but is load‐dependent [23]. The myocardium infiltration of amyloidosis deposits increases myocardial stiffness and altered compliance, leading to a high afterload state at the myocardial level [24]. Therefore, strain values could provide sub‐information about cardiac amyloidosis. Regional strain assessment using speckle‐tracking echocardiography has demonstrated high sensitivity and specificity for diagnosing cardiac amyloidosis and differentiating it from other causes of LV hypertrophy [9]. However, it has been considered for use in clinical setting or on research levels with precautions, because the regional LS exhibits higher variability than the GLS [25].

## 5 Conclusion

The apical sparing phenomenon was more remarkable in TTR‐CM compared with AL‐CM, although both subgroups had a gradual change of strain values from basal to apical segments. RASI might be useful for the discrimination of cardiac amyloidosis subtypes. The relationship of RASI with LV hypertrophy was different between cardiac amyloidosis subtypes, AL‐CM having a significant correlation between RASI and LVMI, but not TTR‐CM. The present study enhances the understanding concerned about the differences in echocardiographic findings between AL‐CM and TTR‐CM.

## Ethics Statement

We obtained approval from the Iinstitutional Ethics Committee of the Tokushima University.

## Conflicts of Interest

The authors declare no conflicts of interest.

## Supporting information



Supporting Information

## Data Availability

The data that support the findings of this study are available on request from the corresponding author. The data are not publicly available due to privacy or ethical restrictions.
