# Supplementary material for: Diagnostic Utility of Relative Apical Sparing Index in Cardiac Amyloidosis Subtypes: A Comparative Study of Immunoglobulin Light Chain and Transthyretin Amyloid Cardiomyopathy
Source: Echocardiography. 2025 Jan 28;42(2):e70087. doi: 10.1111/echo.70087 (PMC11774007; doi:10.1111/echo.70087)
Supplement: Supplementary file 1 — Supporting Information [file ECHO-42-e70087-s001.docx]

**Supplemental Table 1 Clinical characteristics**

|  | **TTR-CM**  **(n = 29)** | **AL -CM**  **(n = 21)** | ***P*** |
| --- | --- | --- | --- |
| **Age, years** | 78 ± 7  (range 64-91) | 66 ± 10  (range 38-80) | <0.001 |
| **Male, %** | 22 (76) | 13 (62) | 0.36 |
| **Body mass index, kg/m^2^** | 1.60 ± 0.19 | 1.60 ± 0.18 | 0.46 |
| **Heart rate, bpm** | 71 ± 14 | 75 ± 11 | 0.17 |
| **Systolic blood pressure, mmHg** | 123 ± 22 | 102 ± 17 | <0.001 |
| **Diastolic blood pressure mmHg** | 71 ± 14 | 63 ± 11 | 0.017 |
| **Hypertension, %** | 7 (24) | 1 (5) | 0.12 |
| **Diabetes mellitus, %** | 2 (7) | 3 (14) | 0.64 |
| **Coronary artery disease, %** | 2 (7) | 0 (0) | 0.50 |
| **White blood cell, ×10^3^/mL** | 6.4 ± 1.7 | 6.1 ± 1.7 | 0.25 |
| **Serum hemoglobin, g/dL** | 14.3 ± 1.8 | 13.2 ± 2.1 | 0.022 |
| **Plate, ×10^5^/ml** | 218 ± 69 | 226 ± 62 | 0.33 |
| **Total serum protein, g/dL** | 7.2 ± 0.5 | 6.0 ± 1.1 | <0.001 |
| **Serum albumin, g/dL** | 4.1 ± 0.4 | 3.3 ± 0.8 | <0.001 |
| **Serum uremia acid, mg/dL** | 6.1 ± 1.5 | 7.0 ± 1.6 | 0.037 |
| **eGFR, mL/min/1.73m^2^** | 53 ± 19 | 53 ± 19 | 0.48 |
| **Creatinine kinase, IU/L** | 160 ± 88 | 111 ± 67 | 0.035 |
| **BNP, pg/mL** | 244 (176-362) | 424 (134-1096) | 0.094 |

Normally distributed data are presented as mean ± SD, whereas non-normally distributed data are presented a median (25^th^-75^th^ percentiles).

*AL-CM*, immunoglobulin light-chain amyloid cardiomyopathy; *ATTR-CM*, transthyretin amyloid cardiomyopathy; *eGFR*, estimated glomerular filtration rate; *BNP*, B-type natriuretic peptide.

**Supplemental** **Table 2 Echocardiographic characteristics**

|  | **TTR -CM**  **(n = 29)** | **AL-CM**  **(n = 21)** | ***P*** |
| --- | --- | --- | --- |
| **IVS wall thickness, mm** | 13.7 ± 2.1 | 12.9 ± 2.2 | 0.25 |
| **Posterior wall thickness, mm** | 13.0 ± 2.2 | 12.5 ± 2.4 | 0.47 |
| **LV mass index, g/m^2^** | 152 ± 39 | 125 ± 49 | 0.015 |
| **Relative wall thickness** | 0.58 ± 0.12 | 0.62 ± 0.14 | 0.17 |
| **Max inferior vena cave, mm** | 13 ± 5 | 13 ± 5 | 0.39 |
| **LV end-diastolic volume, ml** | 93 ± 31 | 78 ± 24 | 0.041 |
| **LV end-systolic volume, ml** | 43 ± 21 | 31 ± 13 | 0.014 |
| **LV ejection fraction, %** | 54 ± 10 | 60 ± 8 | 0.014 |
| **LA volume index, ml/m^2^** | 44 ± 14 | 40 ± 13 | 0.20 |
| **TR-PG, mmHg** | 27 ± 8 | 26 ± 9 | 0.30 |
| **E/A ratio** | 1.75 ± 1.32 | 1.87 ± 1.09 | 0.38 |
| **E/e’ ratio** | 17.7 ± 8.2 | 19.3 ± 7.5 | 0.24 |

Normally distributed data are presented as mean ± SD.

*AL-CM*, immunoglobulin light-chain amyloid cardiomyopathy; *ATTR-CM*, transthyretin amyloid cardiomyopathy; *IVS*, interventricular septum; *LV*, left ventricular; *LA*, left atrial; *TR-PG*, tricuspid regurgitation-pressure gradient.
